# Effects of *Allium mongolicum* regel essential oil supplementation on growth performance, nutrient digestibility, rumen fermentation, and bacterial communities in sheep

**DOI:** 10.3389/fvets.2022.926721

**Published:** 2022-10-31

**Authors:** Zhao Yaxing, Khas Erdene, Bao Zhibi, Ao Changjin, Bai Chen

**Affiliations:** ^1^Inner Mongolia Key Laboratory of Animal Nutrition and Feed Science, College of Animal Science, Inner Mongolia Agricultural University, Hohhot, China; ^2^Animal Husbandry Service Center for Bayannaoer, Bayannaoer, China

**Keywords:** allium mongolicum regel, essential oil, growth performance, rumen fermentation, bacterial communities

## Abstract

The objectives of this research were to investigate the effects of *Allium mongolicum* Regel essential oil on growth performance, nutrient digestibility, rumen fermentation, and bacterial communities in sheep Twenty sheep were randomly divided into two dietary groups with 10 replicates each: (1) a basal diet without AMO as the control group (*n* = 10) and (2) a basal diet supplemented with 40 mg/kg AMO as the AMO group (*n* = 10). The average daily gain (ADG) was increased (*P* < 0.05), and the feed conversion ratio (FCR) was reduced (*P* < 0.05) in the AMO group compared with the control. The ruminal acetate, propionate, total volatile fatty acids (TVFA), and microbial protein (MCP) were higher (*P* < 0.05) in the AMO group than in the control. Moreover, ruminal pH and ammonia nitrogen (NH_3_-N) were lower (*P* < 0.05) in the AMO group than in the control. The relative abundances of the phylum levels of *Firmicutes, Actinobacteriota*, and *Verrucomicrobiota* were higher (*P* < 0.05) in the AMO group than in the control, and the relative abundances of *Bacteroidetes* and *Spirochaetota* were lower (*P* < 0.05) in the AMO group than in the control. The relative abundance of *Prevotella* and *Prevotellaceae_UCG-003* at the genus level was increased (*P* < 0.05) in the AMO group compared with the control; however, the relative abundance of *Succiniclasticum, Norank_f__F082, Christensenellaceae_R-7_group*, and *Norank_f__Muribaculaceae* was decreased (*P* < 0.05) in the AMO group compared with the control. The activities of cellulase, α-amylase, and proteinase were higher (*P* < 0.05) in the AMO group than in the control. The apparent digestibility of dry matter (DM) and crude protein (CP) was increased (*P* < 0.05) in the AMO group compared with the control. In conclusion, AMO supplementation has the potential to improve growth performance. Moreover, supplementation with AMO improved nutrient digestibility, rumen fermentation, and bacterial communities in the rumen of sheep.

## Introduction

Ionophores (monensin) have been extensively used in ruminant production to reduce energy and protein losses in the rumen (1). The appearance of residues in products and the spread of antibiotic-resistant microbes have led to restrictions in the European Union and a ban in China on their use (2, 3). Consequently, the feed industry has investigated safe and effective alternatives that can functionally replace antibiotics, such as plant extracts (phytobiotics) and essential oils (EOs), which provide similar results as antibiotics in terms of feed efficiency and rumen fermentation (4).

EOs are natural bioactive compounds extracted from plants *via* distillation methods, or they can be obtained by chemical extraction (1). EOs as alternatives to antibiotics have the potential to be used in livestock feed. EOs were found to improve nutrient digestibility and increase growth performance in animals (5, 6). Laura et al. noted that a blend of EO dietary supplementation decreased the concentration of acetate and ammonia nitrogen (NH_3_-N) in the rumen of finishing beef cattle (7). Moreover, oregano EO was shown to increase the relative abundance of *Prevotella* and *Dialister* in ruminal fluids *in vitro* (8). This capacity has become a promising research field for investigating the potential of EOs to alter rumen fermentation, resulting in improved feed efficiency in ruminants, but studies have mostly been conducted *in vitro* (9). In small ruminants, only a few *in vivo* studies have been performed to appraise the effects of EOs on animal growth performance, nutrient digestibility, rumen fermentation, and bacterial communities.

The *Allium* genus consists of many species, including shallot (*Allium ascalonicum*), leek (*Allium ampeloprasum*), garlic (*Allium sativum*), onion (*Allium cepa*), and chive (*Allium* schoenoprasum) (10). *Allium mongolicum Regel* (AM) is an *Allium* plant that grows widely in northern China (11). AM is a precious source of forage as it offers extensive nutrients and the contents of protein, ether extract (EE), flavonoids, minerals, amino acids, essential trace elements, and other components (12). A recent study noted that the use of AM water extract could increase the average daily gain (ADG) and decrease the feed conversion ratio (FCR) in sheep (13). In addition, Zhao et al. found that AM ethanol extract at doses of 2.8 g with diets lowered ruminal NH_3_-N values after feeding in sheep (14). AM could increase the dry matter digestibility (DMD) and acid detergent fiber digestibility (ADFD) of Simmental calves (15). According to our previous literature, the use of polysaccharides from AM could decrease the abundances of *f-Prevotellaceae* and *g-Prevotellaceae_UCG-001* in the Bacteroidetes of sheep (16).

To date, no study has assessed the possible impact of *Allium mongolicum* Regel essential oil (AMO) on growth performance, nutrient digestibility, rumen fermentation, and bacterial communities in sheep. The purpose of this study, therefore, was to fill the gap in knowledge in this field and explore the potential of AMO as an antibiotic substitute for sheep.

## Materials and methods

### Ethics approval and consent to participate

All the animal procedures were carried out according to the protocols approved by the College of Animal Science, Inner Mongolia Agricultural University, China. We confirm that the authors complied with the Animal Research Reporting of *in vivo* Experiments (ARRIVE) guidelines.

### Extraction process of AMO

Dried powder derived from AM leaves was purchased from Hao Hai Biological Co., Ltd. (Alashan League, Inner Mongolia, China). AMO was processed from the AM by hydrodistillation (1 kg in 5 L of distilled water) using a Clevenger-type apparatus for 3 h in line with the procedure noted in the European Pharmacopeia (17). The oil acquired above the aqueous distillate was dried with anhydrous sodium sulfate (1 mg/2 mL). The AMO was kept in a stainless sealed container at 4°C until use. AMO was analyzed for its chemical compounds using a GC/MS apparatus at the Atomic and Molecular Physics Unit. The major components of AMO are anethole (37.63%), aromatic hydrocarbon (30.52%), and gingerol (12.60%).

### Animals, diets, and experimental design

Twenty healthy male Dorper sheep with a mean BW (± SD) of 34.5 kg (± 2.5 kg) and an average age of 6 months were used. The sheep were randomly divided into two dietary groups with 10 replicates each: (1) a basal diet without AMO as the control group (*n* = 10) and (2) a basal diet supplemented with 40 mg/kg AMO as the AMO group (*n* = 10). The doses of the AMO were determined based on rumen fermentation using *in vitro* 24-h batch incubation (18). The basal diet was formulated to meet the standard nutrient requirements of sheep according to NRC recommendations (19). The dietary ingredients and nutrient levels of the diet are presented in Table 1. The AMO was completely mixed with the concentrate portion of the TMR before being mixed with the forage component. The diets were provided *ad libitum* twice per day at 07:00 and 18:00. Fresh water was available for the sheep during the whole feeding trial. The experiment lasted 75 days and comprised 15 days for the adaptation period and 60 days for the data and sample collection period.

**Table 1:** Composition and nutrient levels of basal diets of the sheep (DM basis).

**Ingredients**	**Content%**
Alfalfa	27.78
Corn	19.25
Whole corn silage	15.52
Gourd seed skin	12.15
DDGS ^1^	4.33
Flax seed meal	4.62
Sunflower seed meal	6.09
Wheat bran	4.29
Red dates	1.68
Limestone	1.48
CaHPO_4_	0.69
NaCl	0.72
Premix ^2^	1.40
Total	100.00
Nutrient levels ^3^	
DE^4^ (MJ/kg)	16.83
DM%	91.20
CP%	15.32
ADF%	29.69
NDF%	50.98
EE%	3.09
Ca%	1.02
P%	0.55

1 Distillers Dried Grains with Soluble.

2 The premix provided the following per kilogram of diets: 25 mg iron (as FeSO_4_), 29 mg zinc (as ZnSO_4_), 8 mg copper (as CuSO_4_), 30 mg manganese (as MnSO_4_), 0.04 mg iodine (as KI), 0.1 mg cobalt (as CoSO_4_), 3200 IU vitamin A, 1200 IU vitamin D, and 20 IU vitamin E.

3 Digestible energy was a calculated value, whereas the others were measured values.

4 DE, digestible energy; CP, crude protein; ADF, acid detergent fiber; NDF, neutral detergent fiber; EE, ether extract; Ca, calcium; P, phosphorus.

### Data and sample collection

During the trial, sheep were measured for health status, and their body weight was measured at 15-day intervals. Feed was suspended before initial and final weighing. The ADG and dry matter intake (DMI) of sheep were recorded, and the FCR was computed. FCR was determined as average daily DMI/ADG.

The rumen fluid was sampled at 2 h after the morning feeding *via* a stomach tube linked to an Erlenmeyer flask and a vacuum pump on the 60th day of the experimental period. The first 100 mL rumen fluid collected was discarded to prevent saliva contamination, and 100 mL rumen fluid was acquired. pH was immediately determined using an electric handheld pH meter (PHS-3C, Beijing Huoze Experimental Instrument Co., Ltd., Beijing, China). After that, the rumen fluid was then strained immediately through four layers of cheesecloth. One tube of the filtrate with 250 g/L (w/v) metaphosphoric acid (8: 2, v/v) was stored (−20°C) to detect the volatile fatty acids (VFA), and another tube of 10 mL filtrate was preserved by adding 2 mL of 20 g/L (w/v) H_2_SO_4_ for NH_3_-N determination in another tube. The third tube of the filtrate was kept at −20°C to determine the microbial protein (MCP), cellulase, β-glucanase, α-amylase, lipase, and proteinase contents. The fourth tube of the filtrate was stored at – 80°C for analysis of the bacterial composition.

Approximately 100 g of fecal samples was collected from the rectum at 08:00, 11:00, 14:00, and 17:00 on the 58th, 59th, and 60th days of the experimental period to estimate the DMD, organic matter digestibility (OMD), crude protein digestibility (CPD), EE digestibility (EED), neutral detergent fiber digestibility (NDFD), and ADFD. Fecal yield was determined using acid-insoluble ash as an internal indicator (20).

## Sample analysis

### Ruminal NH_3_-N, MCP, and VFA

The VFA contents were measured using gas chromatography (Trace 1100; Thermo Fisher Scientific Co., Ltd., Beijing, China) as described by Zhou et al. (21). The NH_3_-N contents were determined using the method described by the Kjeldahl assay (22). The concentration of MCP yield was determined by urinary purine derivative excretion according to the method described by Hu et al. (23). The cellulase, β-glucanase, α-amylase, lipase, and proteinase activities were analyzed using kits (Nanjing Jiancheng Biological Technology Co. Ltd., Nanjing, China) according to the method of Laura et al. (7).

### DNA extraction and PCR amplification

Microbial community genomic DNA was extracted from ruminal samples using the E.Z.N.A.^®^ soil DNA Kit (Omega Bio-Tek, Norcross, GA, U.S.) according to the manufacturer's instructions. The DNA was extracted according to Jiang (24). The hypervariable region V3-V4 of the bacterial 16S rRNA gene was amplified with the primer pairs 338F (5'-ACTCCTACGGGAGGCAGCAG-3') and 806R (5'-GGACTACHVGGGTWTCTAAT-3') by an ABI GeneAmp^®^ 9700 PCR thermocycler (ABI, CA, USA) (8). The PCR amplification of the 16S rRNA gene was performed according to Liu (25). The PCR mixtures refer to Wang (26). The PCR product was extracted from a 2% agarose gel, purified using the AxyPrep DNA Gel Extraction Kit (Axygen Biosciences, Union City, CA, USA) according to the manufacturer's instructions, and quantified using a Quantus™ Fluorometer (Promega, USA) (26).

### Illumina MiSeq sequencing

Purified amplicons were pooled in equimolar amounts and paired-end sequenced on an Illumina MiSeq PE300 platform/NovaSeq PE250 platform (Illumina, San Diego, USA) according to the standard protocols by Majorbio Bio-Pharm Technology Co. Ltd. (Shanghai, China).

### Processing of sequencing data

The raw 16S rRNA gene sequencing reads were demultiplexed, quality-filtered by fastp version 0.20.0 (27), and merged by FLASH version 1.2.7 (28).

### Feed and fecal chemical analyses

The fecal samples and diets were dried in a forced-air oven at 55°C for 72 h and ground using a 1 mm screen. All feeds were analyzed in duplicate for DM, calcium (Ca), phosphorus (P), EE, CP, and ADF (29). The NDF content was analyzed with sodium sulfite and α-amylase (30). The total fecal output for each sheep was measured based on the content of indigestible neutral detergent fiber (iNDF) and used as an endogenous indicator. The iNDF content was calculated to determine *in vivo* digestibility using the following equation from Menezes et al. (31).

iNDF (% DM) = iNDF (g)/DM (%) × 100

### Statistical analysis

The data were analyzed using SAS software (version 9.0, SAS Inst. Inc., Cary, NC). Before analysis, all data were examined for normality using the UNIVARIATE procedure. Data on growth performance, rumen fermentation parameters, rumen enzyme activities, bacterial relative abundance, and nutrient digestibility were analyzed separately using a one-way analysis of variance (ANOVA) with dietary treatments used as the main factor.

Statistically significant differences among treatments were evaluated using Tukey's multiple range test. Probability values of *P* ≤ 0.05 were declared significant, and probability values were declared statistical trends when 0.05 < *P* ≤ 0.10.

## Results

### Effects of AMO on growth performance and feed efficiency

The effects of AMO on the growth performance and feed efficiency of sheep are shown in Table 2. Dietary supplementation with AMO increased (*P* < 0.05) ADG compared with the control. Dietary supplementation with AMO decreased (*P* < 0.05) FCR compared with the control. There was no effect of AMO inclusion in the diet on DMI (*P* > 0.05).

**Table 2:** Effects of AMO supplementation in sheep diet on growth performance.

**Items^2^**	**Treatments** ^**1**^		**SEM^3^**	***P*-value**
	**CON**	**AMO**		
Initial body weight	34.23	35.19	0.734	0.376
Final body weight	46.55	48.62	3.103	0.535
ADG / (kg/d)	0.167	0.178	0.004	0.043
DMI / (g/d)	1380.66	1406.67	25.353	0.363
FCR	8.27	7.89	0.077	0.005

^1^Treatments: CON, basal diet; AMO, *Allium mongolicum* Regel essential oil.

^2^DMI, dry matter intake; ADG, average daily gain; FCR, feed conversion ratio.

^3^Standard error of mean.

### Effects of AMO on rumen fermentation parameters

The effects of AMO on the rumen fermentation parameters of sheep are shown in Table 3. Dietary supplementation with AMO increased (*P* < 0.05) the concentrations of MCP, TVFA, acetate, and propionate compared with the control. Moreover, a lower rumen pH (*P* < 0.05) and a lower NH_3_-N (*P* < 0.05) concentration were noted in the AMO group. There were no effects of AMO inclusion in the diet on the acetate-to-propionate ratio or the concentrations of butyrate, isobutyrate, valerate, and isovalerate (*P* > 0.05).

**Table 3:** Effect of AMO on ruminal fermentation in sheep.

**Items^2^**	**Treatments** ^**1**^		**SEM^3^**	***P*-value**
	**CON**	**AMO**		
pH	6.61	6.49	0.026	0.010
NH_3_-N mg/dL	18.83	16.80	0.272	0.005
TVFA mmol/L	97.13	100.76	1.054	0.034
MCP mg/dL	163.66	175.65	3.986	0.040
Acetate mmol/L	59.37	63.66	0.909	0.016
Propionate mmol/L	23.07	25.33	0.625	0.037
Butyrate mmol/L	10.94	9.10	2.285	0.387
Isobutyrate mmol/L	1.37	1.46	0.082	0.109
Valerate mmol/L	1.27	1.22	2.049	0.570
Isovalerate mmol/L	1.13	1.20	3.029	0.061
A/P	2.57	2.51	0.070	0.459

^1^Treatments: CON, basal diet; AMO, *Allium mongolicum* Regel essential oil.

^2^NH_3_-N, ammonia nitrogen; TVFA, total VFA; MCP, microbial protein; A/P, Acetate/Propionate.

^3^Standard error of mean.

### Effects of AMO on the rumen bacterial community

Table 4 shows that the OTU (Operational Taxonomic Unit), Shannon, Simpson, Ace, Chao1, and coverage indices were not affected (*P* > 0.05) by AMO. Table 5 shows that the relative abundance of the phylum level *Bacteroidetes* and *Spirochaetota* in the AMO group was lower than that in the control. Conversely, the relative abundances of *Firmicutes, Actinobacteriota*, and *Verrucomicrobiota* of sheep fed the AMO diet were higher (*P* < 0.05) than those fed the control diet. Furthermore, no differences were noted for the relative abundances of other rumen bacterial phyla between treatments.

**Table 4:** Effect of AMO on rumen bacterial diversity.

**Items**	**Treatments** ^**1**^		**SEM^2^**	***P*-value**
	**CON**	**AMO**		
OTU^3^	1417	1452	18.70	0.179
Shannon	4.88	5.14	0.169	0.268
Simpson	0.03	0.02	0.01	0.770
Ace	1057.66	1155.32	38.32	0.060
Chao1	1079.29	1169.51	60.31	0.273
Coverage	0.95	0.96	0.01	0.770

^1^Treatments: CON, basal diet; AMO, *Allium mongolicum* Regel essential oil.

^2^Standard error of mean.

^3^OTU, Operational Taxonomic Unit.

**Table 5:** Effect of AMO on rumen bacterial composition at phylum level (%).

**Items**	**Treatments** ^**1**^		**SEM^2^**	***P*-value**
	**CON**	**AMO**		
*Bacteroidetes*	50.50	58.45	0.859	0.001
*Firmicutes*	45.12	37.65	0.722	0.001
*Actinobacteriota*	1.52	0.71	0.084	0.001
*Desulfobacterota*	0.65	0.66	0.056	0.827
*Spirochaetota*	0.27	0.85	0.049	0.000
*Verrucomicrobiota*	0.43	0.23	0.018	0.001
*Synergistota*	0.33	0.31	0.054	0.690
*Patescibacteria*	0.35	0.37	0.047	0.743
*Unclassified_k_norank_d_bacteria*	0.34	0.33	0.029	0.754
*Chloroflex*	0.08	0.08	0.010	0.770

^1^Treatments: CON, basal diet; AMO, *Allium mongolicum* Regel essential oil.

^2^Standard error of mean.

Table 6 shows that the relative abundances of the genera *Prevotella* (*P* < 0.05) and *Prevotellaceae_UCG-003* (*P* < 0.05) were increased in the AMO group compared with those in the control. In contrast, sheep fed the AMO diet decreased the relative abundance of *Succiniclasticum, norank_f__F082, Christensenellaceae_R-7_group*, and *norank_f__Muribaculaceae* (*P* < 0.05). Moreover, no differences were noted for the relative abundances of other rumen bacterial genera between treatments.

**Table 6:** Effect of AMO on rumen bacterial composition (%) at genus level.

**Items**	**Treatments** ^**1**^		**SEM^2^**	***P-*value**
	**CON**	**AMO**		
*Prevotella*	15.82	26.20	1.24	0.004
*Rikenellaceae_RC9_gut_group*	17.67	16.59	0.736	0.218
*Succiniclasticum*	7.51	3.85	0.40	0.007
*Norank_f__F082*	6.68	4.70	0.172	0.006
*Christensenellaceae_R-7_group*	4.71	4.42	0.049	0.015
*Norank_f__Muribaculaceae*	3.63	2.40	0.317	0.033
*NK4A214_group*	2.74	2.62	0.144	0.432
*Norank_f__UCG-011*	2.85	2.75	0.098	0.355
*Prevotellaceae_UCG-003*	1.99	2.66	0.074	0.004
*Quinella*	2.79	2.72	0.355	0.846
*Norank_f__Bacteroidales_UCG-001*	2.02	2.10	0.104	0.519

^1^Treatments: CON = basal diet; AMO = *Allium mongolicum* Regel essential oil.

^2^Standard error of mean.

### Effects of AMO on rumen enzyme activities

The effects of AMO on the rumen enzyme activities of sheep are shown in Table 7. Dietary supplementation with AMO increased (*P* < 0.05) the activities of cellulase, α-amylase, and proteinase. In addition, no difference was found for the activities of rumen lipase and β-glucanase (*P* > 0.05).

**Table 7:** Effect of AMO on enzyme activities in sheep.

**Items**	**Treatments** ^**1**^		**SEM^2^**	***P*-value**
	**CON**	**AMO**		
Cellulase (U/L)	419.82	477.68	6.140	0.000
β-glucanase (U/mL)	6.30	6.36	0.217	0.484
α-amylase (U/dL)	47.92	59.83	0.897	0.000
Lipase (U/L)	87.59	91.80	2.664	0.145
Proteinase (U/dL)	80.34	83.10	0.913	0.024

^1^Treatments: CON, basal diet; AMO, *Allium mongolicum* Regel essential oil.

^2^Standard error of mean.

### Effects of AMO on nutrient digestibility

The effects of AMO on the apparent digestibility of sheep are shown in Table 8. Dietary supplementation with AMO increased (*P* < 0.05) the apparent digestibility of DM and CP. No difference was found for the apparent digestibility of OM, EE, ADF, and NDF (*P* > 0.05).

**Table 8:** Effect of AMO on nutrient digestibility in sheep (%).

**Items^2^**	**Treatments** ^**1**^		**SEM^3^**	***P*-value**
	**CON**	**AMO**		
DMD	66.51	69.66	0.907	0.026
OMD	67.46	69.39	0.843	0.084
CPD	61.58	63.43	0.459	0.016
EED	80.53	79.23	0.625	0.105
NDFD	45.8	45.92	0.799	0.891
ADFD	27.04	25.95	0.701	0.195

^1^Treatments: CON, basal diet; AMO, *Allium mongolicum* Regel essential oil.

^2^DMD, dry matter digestibility; OMD, organic matter digestibility; CPD, crude protein digestibility; EED, ether extract digestibility; NDFD, neutral detergent fiber digestibility; ADFD, acid detergent fiber digestibility.

^3^Standard error of mean.

## Discussion

### Effects of AMO on growth performance and feed efficiency

As one of the main components of AM, AM flavonoids have been used as feed additives to increase the growth hormone (GH) level in the serum and ADG of sheep (32). Du et al. reported an improvement in the growth performance of sheep with dietary inclusion of AM extract (3.4 g/sheep/day) (16). However, scarce literature is available with regard to the effects of dietary supplementation with AMO on the DMI and ADG of sheep. The present research investigated whether sheep-fed AMO had a change in DMI compared with the control group. However, in contrast to this finding, an enhancement in ADG was found for sheep fed the diet supplemented with AMO. In the current study, the addition of AMO to the diet of sheep clearly decreased FCR, resulting from the increased ADG and similar DMI. These results are consistent with Zhao et al., who stated that diets supplemented with AM water extract in sheep resulted in changes in ADG and FCR (14). Such favorable effects on ADG and FCR were likely due to an increase in the relative abundance of bacteria involved in carbohydrate metabolism in the rumen, thereby enhancing the digestibility of DM and CP. Furthermore, the increases in growth performance may be partly related to the immunoenhancement and antioxidative effects of AMO (33).

### Effects of AMO on rumen fermentation parameters

The decrease in ruminal Ph following AMO supplementation was in accordance with the increase in TVFA content (34). A lower ruminal pH was reported in sheep receiving AMO addition and was 6.49, which was suitable for rumen microbial growth and nutrient degradation. The major products of carbohydrate fermentation in the rumen are VFAs, which are the primary energy sources of ruminants (24). The total and molar proportions of VFA generated in the rumen depend on the substrate composition, nutrient availability, ruminal microbial community, and rumen pH reached (35). These alterations in TVFA proportions could be due to the stimulating action of AMO on propionate- and acetate-producing bacteria, indicating that AMO increased the fermentation of carbohydrates. It was also noted that AM water extract and AM ethanol extract could increase the quantity of amylolytic bacteria and cellulolytic bacteria in the rumen (14). This could be used for carbohydrate fermentation, generating high VFA. These current results differ in part from those of Ding et al., who did not observe a difference in the ruminal concentrations of TVFA or acetic and propionic acids in the rumen fluid of sheep-fed AM diets (36). Zhao et al. also found that feeding sheep with AM water extract increased the content of butyric acid and isobutyric acid (14). The difference between studies could be related to the source of AMO and AM water extract, the amount consumed, and the basic diet.

Our previous research found that the use of AMO decreases the content of NH_3_-N in *in vitro* rumen fermentation (18). Consistent with the above study, the addition of AMO decreased the NH_3_-N content in the rumen of sheep. Wallace et al. affirmed that a rumen NH_3_-N content between 15 and 30 mg/dL was more reasonable (37). Consequently, we reported that using AMO as a dietary additive could accelerate nitrogen metabolism in the rumen within a legitimate range (16.80 mg/dL) in the present study. MCP is synthesized by rumen microorganisms using NH_3_-N, peptide, and amino acids in the rumen, and energy is necessary during the whole process of MCP synthesis (24). The contents of MCP and NH_3_-N maintain a balance in the rumen, and excessive NH_3_-N will increase the loss of nitrogen in the nitrogen cycle, while less NH_3_-N will influence the production of MCP (24). The present research found that the output of MCP in the rumen was increased by feeding a diet with AMO to sheep, which indicates that AMO addition supplies an adequate nitrogen source, carbon skeleton, and energy for MCP synthesis. A similar consequence was acquired with dietary supplementation with different levels of AMO in the diet, which enhanced the rumen MCP yield *in vitro* (18).

### Effects of AMO on the rumen bacterial community

The rumen is colonized by some microorganism communities (38), which play significant roles in fermenting and digesting nutrients to supply protein and energy resources (39), improving resistance to external stress, and enhancing growth and performance (40). The most significant microorganisms in the rumen were bacteria, as were also mentioned in previous literature (41). These bacterial communities live in dynamic conditions that could be influenced by several factors, such as diet, animal age, and feed additives (42). In the current study, there was no significant difference in OTU, Ace, Chao1, Simpson, Shannon, and coverage, which suggested that the addition of AMO had no negative effect on the alpha diversity of rumen microbes.

In the present research, *Bacteroidetes* and *Firmicutes* were the superior phyla in the ruminal microbial community, which is in accordance with previous literature in sheep (43, 44). In the current study, AMO addition in diets increased the relative abundance of the phylum *Firmicutes* and decreased the relative abundance of the phylum *Bacteroidetes*, consequently increasing the *Firmicutes* to *Bacteroidetes* (F:B) ratio, which could be beneficial for energy utilization and growth performance (45). The increased F:B ratio might be due to the acceleration of growth of *Firmicutes* by the antimicrobial function of the active ingredient in AMO.

In ruminants, *Prevotella* is the genus of the phylum *Bacteroidetes* with the highest relative abundance (26). *Prevotella* plays an important role in secreting the enzyme xylanase, which contributes to polysaccharide degradation (46). Some species of *Prevotella* are involved in the degradation and utilization processes of fiber, hemicellulose, pectin, and protein (47). In agreement with these results, our study found that the relative abundance of *Prevotella* seemed to be the most abundant in all rumen fluid samples at the genus level. Furthermore, the current work also found that the use of AMO in the sheep diet could promote the relative abundance of *Prevotellaceae_UCG-003*. The connection between the substrate and growth of *Prevotella* is not apparent. Previous studies have confirmed that *Prevotellaceae UCG-003* participates in glucose metabolism, generating acetic acids as the primary fermentation final products (25). This explains why acetic acid was present at a higher level in the AMO group.

### Effects of AMO on enzyme activity

Meanwhile, the rumen cellulase, α-amylase, and proteinase activity apparently increased in the AMO group compared with the control. This could be sensed by a higher rumen *Prevotella* and *Prevotellaceae_UCG-003* in the AMO group, which contribute to the production of cellulase, α-amylase, and proteinase. Similar to the results observed in this study by Zhao et al., AM extracts could enhance the activities of cellulase, α-amylase, and proteinase in the rumen (14).

### Effects of AMO on nutrient digestibility

Because AMO has been observed to decrease NH_3_-N accumulation and change microbial community structure, we supposed that AMO would enhance nutrient digestibility in sheep by improving rumen microbial synthesis efficiency. Qiao et al. suggested that dietary AMO addition promoted the apparent total tract digestibility of CP in sheep (48). In addition, Yaxing et al. (33) noted increased DM digestibility in *in vitro* batch cultures with rumen fluid of sheep supplemented with increasing levels of AMO. Data from our research found that the digestibility of DM and CP in sheep with AMO supplementation was increased, which was similar to what we hypothesized. Similarly, several studies have reported that nutrient digestibility is enhanced by cinnamon oil in chickens (49), by oregano essential oil in lambs (8), and by essential oil in beef cattle (50). There are three reasons for the results of the present study. First, the decreased ruminal NH_3_-N with sheep AMO inclusion suggested that the synthesis of MCP had accelerated and led to the increased CP digestibility. Second, Dowd et al. observed that *Prevotella* was very significant for the degradation and absorption of proteins as well as for the fermentation of peptides in the rumen (51). Based on the above findings, our present research detected that the relative abundance of *Prevotella* was most abundant in the rumen fluid of sheep in the two treatments at the genus level. Moreover, the current research also demonstrated that supplementation with AMO in the diet could increase the relative abundance of *Prevotella* and *Prevotellaceae_UCG-003*. These results revealed that dietary addition of AMO had a favorable effect on enhancing the degradation of CP in the rumen, thereby boosting rumen fermentation. Third, we surmised that the improved digestibility of DM and CP was partially due to the elevated ruminal proteinase enzyme activity.

## Conclusion

In general, supplementation with AMO in sheep diets increased growth performance by increasing ADG and decreasing FCR, promoted rumen fermentation, diversified the bacterial community composition in the rumen, enhanced the apparent digestibility of DM and CP, and improved rumen enzyme activities.

## Data availability statement

The datasets presented in this study can be found in online repositories. The names of the repository/repositories and accession number(s) can be found below: NCBI; PRJNA833585.

## Ethics statement

The animal study was reviewed and approved by College of Animal Science, Inner Mongolia Agricultural University, China. Written informed consent was obtained from the owners for the participation of their animals in this study.

## Author contributions

ZY wrote the main manuscript. KE and BZ prepared Tables 1–4, and BC prepared Tables 5–8. AC edited the manuscript. All authors have read and agreed to the published version of the manuscript.

## Funding

This study was funded by the landmark achievement of Inner Mongolia Agricultural University (BZCG202102).

## Conflict of interest

The authors declare that the research was conducted in the absence of any commercial or financial relationships that could be construed as a potential conflict of interest.

## Publisher's note

All claims expressed in this article are solely those of the authors and do not necessarily represent those of their affiliated organizations, or those of the publisher, the editors and the reviewers. Any product that may be evaluated in this article, or claim that may be made by its manufacturer, is not guaranteed or endorsed by the publisher.

## References

[1] EI-essawy AM Anele UY Abdel-Wahed AM Abdou AR Khattab IM. Effects of anise, clove and thyme essential oils supplementation on rumen fermentation, blood metabolites, milk yield and milk composition in lactating goats. Animal Feed Sci Technol. (2020) 10:114760. 10.1016/j.anifeedsci.2020.114760

[2] Magnusson U. Prudent and effective antimicrobial use in adiverse livestock and consumer's world. J. Anim. Sci. (2020) 98:S4–S8. 10.1093/jas/skaa148 32810240 PMC7531223

[3] Guo S Lei J Liu L Qu X Li P Liu X. Effects of Macleaya cordata extract on laying performance, egg quality and serum indices in Xuefeng Black-Bone Chicken. Poult Sci. (2021) 100[4]:101031. 10.1016/j.psj.2021.101031 33684648 PMC7938252

[4] Aziz MS. Karboune S. Natural antimicrobial/antioxidant agents in meat and poultry products as well as fruits and vegetables: a review. Crit Rev Food Sci Nutr. (2018) 58:486–511. 10.1080/10408398.2016.1194256 27437876

[5] Ruan D Fan Q Fouad AM Sun Y Huang S Wu A. Effects of dietary oregano essential oil supplementation on growth performance, intestinal antioxidative capacity, immunity and intestinal microbiota in yellow-feathered chickens. J Animal Sci. (2021) 99:1–11. 10.1093/jas/skab033 33544855 PMC7918158

[6] Parvar R Ghoorchi T Kashfi H Parvar K. Effect of Ferulago angulata (Chavil) essential oil supplementation on lamb growth performance and meat quality characteristics. Small Ruminant Res. (2018) 7:26. 10.1016/j.smallrumres.2018.07.026

[7] Toseti LB Goulart RS Gouvêa VN Acedo TS Vasconcellos GS Pires AV. Effects of a blend of essential oils and exogenous α- amylase in diets containing different roughage sources for finishing beef cattle. Animal Feed Sci Technol. (2020) 269:114643. 10.1016/j.anifeedsci.2020.114643

[8] Zhou R Wu J Lang X Liu L Casper DP Wang C. Effects of oregano essential oil on *in vitro* ruminal fermentation, methane production, and ruminal microbial community. J Dairy Sci. (2020) 103:2303–14. 10.3168/jds.2019-16611 31954586

[9] Joch M Kudrna V Hakl J Božik M Homolka P Illek J. *In vitro* and *in vivo* potential of a blend of essential oil compounds to improve rumen fermentation and performance of dairy cows. Animal Feed Sci Technol. (2019) 3:009. 10.1016/j.anifeedsci.2019.03.009

[10] Zeng Y Li Y Yang J Pu X Du J Yang X. Therapeutic role of functional components in alliums for preventive chronic disease in human being. Evid. Based Complem. Altern. Med. (2017) 94:02849. 10.1155/2017/9402849 28261311 PMC5316450

[11] Schmitt B Schulz H Storsberg J Keusgen M. Chemical characterization of Allium ursinum L. depending on harvesting time. J Agric Food Chem. (2005) 53:e94. 10.1021/jf0504768 16131144

[12] Huang QC Chu YL Zhao YH Zhao HT Liu JM Zhou RY. Status and prospect of plant fiber industry in Guangxi province. Plan Fib Sci. (2011) 33:202–5. 10.3969/j.issn.1671-3532.2011.04.010

[13] Yaxing Z Erdene K Changjin A Zhibi B Hongxi D Zejun F. Effects of Allium mongolicum Regel and its extracts supplementation on the growth performance, carcass parameters and meat quality of sheep. Italian J Animal Sci. (2021) 20:1899–1908. 10.1080/1828051X.2021.1971572

[14] Zhao Y Ao C Bao Z Fan Z Liu W Ding H. Effects of Allium monogolium Regel and its extracts on rumen fermentation and microflora of sheep. Anim Nutr. (2019) 31:2313–22. 10.3969/j.issn.1006-267x.2019.05.038

[15] Xie K Wang Z Wang Y Wang C Chang S Zhang C. Effects of Allium mongolicum Regel supplementation on the digestibility, methane production, and antioxidant capacity of Simmental calves in northwest China. Animal Sci J. (2020) 91:e13392. 10.1111/asj.13392 32557991

[16] Du H Khas E Chen S Qi S Bao Z Zhao Y. Correlation of the rumen fluid microbiome and the average daily gain with a dietary supplementation of allium mongolicum regel extracts in sheep. J Anim Sci. (2019) 97:2865–77. 10.1093/jas/skz139 31074483 PMC6606504

[17] Egyptian Pharmacopoeia (EP) General Organization for Governmental Printing Affairs Cairo Egypt (2005).

[18] Mu CT Ding N Hao XY Zhao YB Wang PJ Zhao JX. Effects of Allium mongolicum Regel essential oil on *in vitro* rumen fermentation and substrate degradability of sheep. Anim Nutr. (2021) 33:4740–47. 10.3969/j.issn.1006-267x.2021.08.052

[19] NRC. Nutrient requirements of sheep. 11th rev. edn. Washington, DC: National Academic Press (2012).

[20] Van Keulen J Young BA. Evaluation of acid-insoluble ash as a natural marker in ruminant digestibility studies. J. Anim. Sci. (1977) 44:282–289. 10.2527/jas1977.442282x

[21] Zhou JW Mi JD Degen AA Guo XS Wang HC Ding LM. Apparent digestibility, rumen fermentation and nitrogen balance in Tibetan and fine-wool sheep offered forage-concentrate diets differing in nitrogen concentration. J. Agric. Sci. (2015) 153:1135–45. 10.1017/S0021859615000465

[22] Broderick GA Kang JH. Automated simultaneous determination of ammonia and total amino acids in ruminal fluid and *in vitro* media. J. Dairy Sci. (1980) 63:64–75. 10.3168/jds.S0022-0302(80)82888-8 7372898

[23] Hu WL Liu JX Ye JA Wu YM Guo YQ. Effect of tea saponin on rumen fermentation *in vitro*. Anim Feed Sci Technol. (2005) 120:333–9. 10.1016/j.anifeedsci.2005.02.029

[24] Jiang X Liu X Liu S Li Y Zhao HB Zhang YG. Growth, rumen fermentation and plasma metabolites of Holstein male calves fed fermented corn gluten meal during the postweaning stage. Animal Feed Sci Technol. (2019) 249:1–9. 10.1016/j.anifeedsci.2019.01.012

[25] Liu YZ Chen X Zhao W Lang M Zhang XF Wang T. Effects of yeast culture supplementation and the ratio of non-structural carbohydrate to fat on rumen fermentation parameters and bacterial-community composition in sheep. Animal Feed Sci Technol. (2019) 2:3. 10.1016/j.anifeedsci.2019.02.003

[26] Wang T Jiao J Wang H Degen AA Gou N Li S. The effects of supplementing sweet sorghum with grapeseeds on dry matter intake, average daily gain, feed digestibility and rumen parameters and microbiota in lambs. Animal Feed Sci Technol. (2020) 14:114750. 10.1016/j.anifeedsci.2020.114750

[27] Chen S Zhou Y Chen Y Gu J. Fastp: an ultra-fast all-in-one FASTQ. Preprocessor. Bioinformatics. (2018) 34:i884–90. 10.1093/bioinformatics/bty560 30423086 PMC6129281

[28] Magoč T Salzberg SL. FLASH: fast length adjustment of short reads to improve genome assemblies. Bioinformatics. (2011) 27:2957–63. 10.1093/bioinformatics/btr507 21903629 PMC3198573

[29] Association of Official Analytical Chemists (AOAC). Changes in official methods of analysis of the Association of Official Analytical Chemists. Arlington. (1990) 110:123–35.

[30] Van Soest PJ Robertson JB. Lewis BA. Methods for dietary fiber, neutral detergent fiber, and nonstarch polysaccharides in relation to animal nutrition. J Dairy Sci. (1991) 74:3583–97. 10.3168/jds.S0022-0302(91)78551-2 1660498

[31] Menezes AC Valadares Filho SC e Silva LC Pacheco MV Pereira JM Rotta PP. Does a reduction in dietary crude protein content affect performance, nutrient requirements, nitrogen losses, and methane emissions in finishing Nellore bulls? Agric Ecosyst Environ. (2016) 3:015. 10.1016/j.agee.2016.03.015

[32] Ren WC Cui FW. Chang JA. Effects of flavonoids from Allium mongolicum Regel on growth performance and growth-related hormones in meat sheep. Anim Nutr. (2017) 3:33–8. 10.1016/j.aninu.2017.01.003 29767126 PMC5941079

[33] Yaxing Z Erdene K Changjin A Zhibi B Hongxi D Zejun F. Effects of essential oil from Allium mongolicum Regel on slaughter performance, physicochemical property of meat and Antioxidant activity in mutton sheep. Feed Ind. (2021) 42:60–64. 10.13302/j.cnki.fi.2021.17.011

[34] Dijkstra J Ellis JL Kebreab E Strathe AB L'opez S France J Bannink A. Ruminal pH regulation and nutritional consequences of low pH. Anim. Feed. Sci. Tech. (2012) 172:22–33. 10.1016/j.anifeedsci.2011.12.005

[35] Ceconi I Ruiz-Moreno MJ DiLorenzo N DiCostanzo A Crawford GI. Effect of urea inclusion in diets containing corn dried distillers grains on feedlot cattle performance, carcass characteristics, ruminal fermentation, total tract digestibility, and purine derivatives-to-creatinine index. J. Anim. Sci. (2015) 93:357–369. 10.2527/jas.2014-8214 25412747

[36] Ding H Liu W Ao C Li S Li Y Zhang Z. Effects of dietary allium mongolicum regel powder or probiotic complex preparation on ruminal fermentation parameters and rumen fluid bacterial diversity of dorper × thin-tailed han crossbred sheep. Anim Nutr. (2018) 31:324–33. 10.3969/j.issn.1006-267x.2019.01.039

[37] Wallace RJ Rooke JA McKain N Duthie CA Hyslop JJ Ross DW. Effect of ruminal NH [[sb]]3[[/s]] -N levels on ruminal fermentation, purine derivatives, digestibility and rice straw intake in swamp buffaloes. Asian-Australas. J. Anim. Sci. (1999) 12:904–907. 10.5713/ajas.1999.904

[38] Zhang RY Jin W Feng PF Liu JH Mao SY. High-grain diet feeding altered the composition and functions of the rumen bacterial community and caused the damage to the laminar tissues of goats. Animal. (2018) 12:1–10. 10.1017/S175173111800040X 29553005

[39] Vymazal J Kröpfelová L. Ruminal microbiota developing in different *in vitro* simulation systems inoculated with goats' rumen liquor. Anim. Feed Sci. Technol. (2013) 185:9–18. 10.1016/j.anifeedsci.2013.06.003

[40] Cani P D Delzenne NM. The role of the gut microbiota in energy metabolism and metabolic disease. Curr. Pharm. Design. (2009) 15:1546–58. 10.2174/138161209788168164 19442172

[41] Wang YY Cao PH Wang L Zhao ZY Chen YL Yang YX. Bacterial community diversity associated with different levels of dietary nutrition in the rumen of sheep. Appl. Microbiol. Biot. (2017) 101:3717–28. 10.1007/s00253-017-8144-5 28175950

[42] O'Hara E Kenny DA McGovern E Byrne CJ McCabe MS Guan LL. Investigating temporal microbial dynamics in the rumen of beef calves raised on two farms during early life. FEMS Microbiol. Ecol. (2020) 96:114802. 10.1093/femsec/fiz203 31917419

[43] Trabi EB Seddik H Xie F Wang X Liu J Mao S. Effect of pelleted high-grain total mixed ration on rumen morphology, epithelium-associated microbiota and gene expression of proinflammatory cytokines and tight junction proteins in Hu sheep. Anim. Feed Sci. Technol. (2020) 263:114453. 10.1016/j.anifeedsci.2020.114453

[44] Xia G Sun J Fan J Zhao F Ahmed G Jin Y Zhang Y Wang H. β-sitosterol attenuates high grain diet-induced inflammatory stress and modifies rumen fermentation and microbiota in Sheep. Animals. (2020) 10:171. 10.3390/ani10010171 31963945 PMC7022687

[45] Thomas M Webb M Ghimire S Blair A Olson K Fenske GJ. Metagenomic characterization of the effect of feed additives on the gut microbiome and antibiotic resistome of feedlot cattle. Sci. Rep. (2017) 7:12257. 10.1038/s41598-017-12481-6 28947833 PMC5612972

[46] Kim M Morrison M Yu ZT. Status of the phylogenetic diversity census of ruminal microbiomes. FEMS Microbiol. Ecol. (2011) 76:49–63. 10.1111/j.1574-6941.2010.01029.x 21223325

[47] Wei Y Zhao CX Li XW Cong LX Zhang H Liu GW. Effect of different crude fiber source diets on rumen microflora of sika deer. Chin. J. Vet. Sci. (2018) 38:217–21. 10.16303/j.cnki.1005-4545.2018.01.35

[48] Qiao YJ. Effects of adding allium mongolicum regel essential oil in diets on production performance, apparent digestibility of nutrients and small intestine morphology and structure of mutton sheep. Master's Thesis. (2021) 43:24–29. 10.13302/j.cnki.fi.2021.11.001

[49] Chowdhury Subrata Mandal Guru P Patra Amlan K. Different essential oils in diets of chickens: 1.Growth performance, nutrient utilization, nitrogen excretion, carcass traits and chemical composition of meat. Animal Feed Sci Technol. (2017) 12:2. 10.1016/j.anifeedsci.2017.12.002

[50] Torres RN Paschoaloto JR Ezequiel JM da Silva DA Almeida MT. Meta-analysis of the effects of essential oil as an alternative to monensin in diets for beef cattle. The Veterinary Journal. (2021) 272:105659. 10.1016/j.tvjl.2021.105659 33941330

[51] Dowd SE Callaway TR Wolcott RD Sun Y McKeehan T Hagevoort RG. Evaluation of the bacterial diversity in the feces of cattle using 16S rDNA bacterial tag-encoded FLX amplicon pyrosequencing (bTEFAP). BMC Microbiol. (2008) 8:125. 10.1186/1471-2180-8-125 18652685 PMC2515157

