# Supplementary figures and images for: Effects of Allium mongolicum regel essential oil supplementation on growth performance, nutrient digestibility, rumen fermentation, and bacterial communities in sheep
Source: Front Vet Sci. 2022 Oct 31;9:926721. doi: 10.3389/fvets.2022.926721 (PMC9659749; doi:10.3389/fvets.2022.926721)

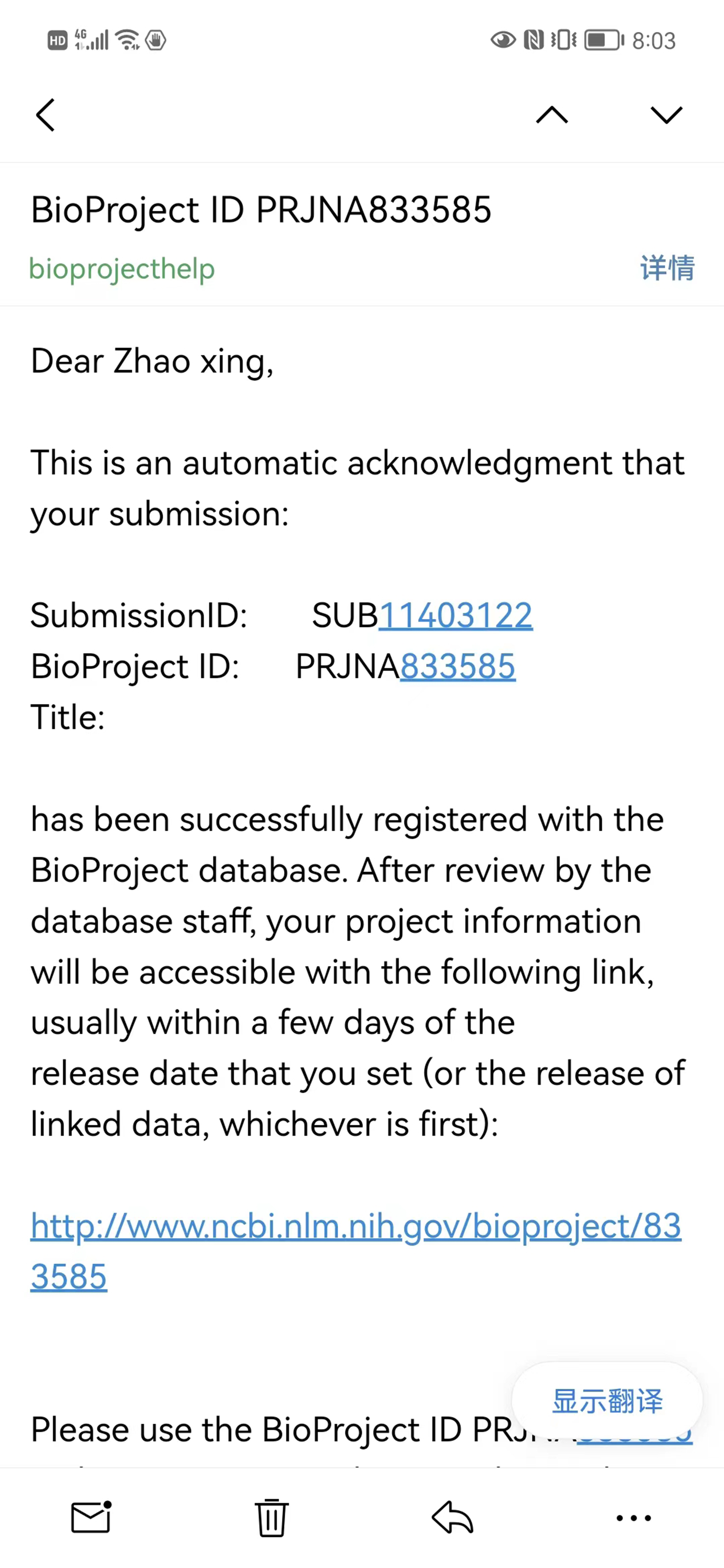

Supplement: Supplementary file 1 [file Image_1.JPEG]
